# Supplementary material for: The causal role of circulating inflammatory markers in osteoporosis: a bidirectional Mendelian randomized study
Source: Front Immunol. 2024 Jul 18;15:1412298. doi: 10.3389/fimmu.2024.1412298 (PMC11291241; doi:10.3389/fimmu.2024.1412298)
Supplement: Supplementary file 1 [file DataSheet_1.zip › supplementary material/OSTEOPOROSIS-CIM/GCST90274828.tsv.gz_leave_one_out.pdf]

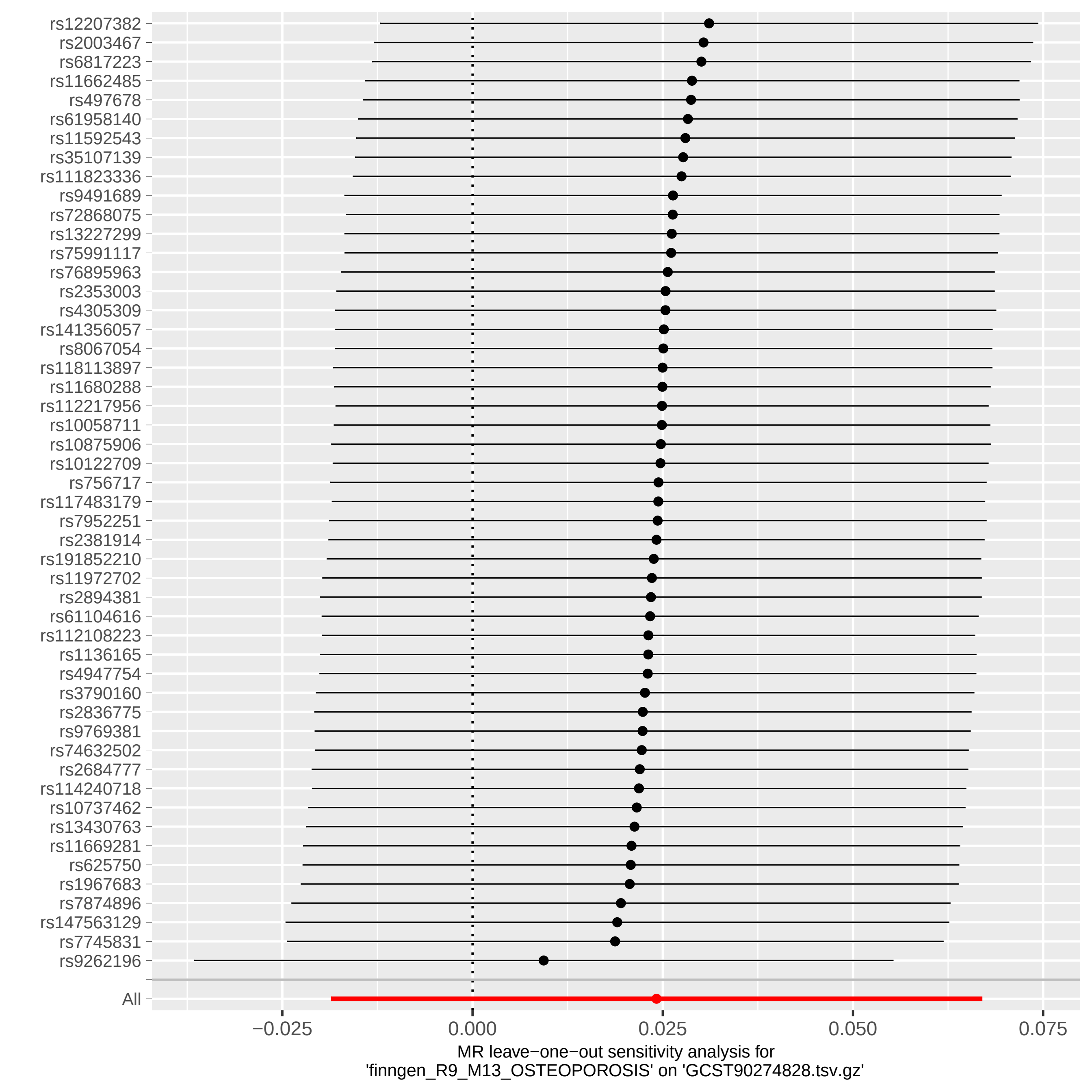
rs12207382
rs2003467
rs6817223
rs11662485
rs497678
rs61958140
rs11592543
rs35107139
rs111823336
rs9491689
rs72868075
rs13227299
rs75991117
rs76895963
rs2353003
rs4305309
rs141356057
rs8067054
rs118113897
rs11680288
rs112217956
rs10058711
rs10875906
rs10122709
rs756717
rs117483179
rs7952251
rs2381914
rs191852210
rs11972702
rs2894381
rs61104616
rs112108223
rs1136165
rs4947754
rs3790160
rs2836775
rs9769381
rs74632502
rs2684777
rs114240718
rs10737462
rs13430763
rs11669281
rs625750
rs1967683
rs7874896
rs147563129
rs7745831
rs9262196
All
−0.025
0.000
0.025
0.050
0.075
MR leave−one−out sensitivity analysis for
'finngen_R9_M13_OSTEOPOROSIS' on 'GCST90274828.tsv.gz'
